# MicroPure Imaging for the Evaluation of Microcalcifications in Gouty Arthritis Involving the First Metatarsophalangeal Joint: *A Preliminary Study*

**DOI:** 10.1371/journal.pone.0095743

**Published:** 2014-05-02

**Authors:** Lu Yin, Jiaan Zhu, Qin Xue, Niansong Wang, Zhenlong Hu, Yunxia Huang, Fang Liu, Bing Hu

**Affiliations:** 1 Department of Ultrasound, Shanghai Jiao Tong University Affiliated Sixth People's Hospital, Shanghai Institute of Ultrasound in Medicine, Shanghai, China; 2 Department of Rheumatology, Shanghai Jiao Tong University Affiliated Sixth People's Hospital. Shanghai, China; Center for Rheumatic Diseases, India

## Abstract

**Objective:**

To assess the value of MicroPure, a new ultrasound image processing technique, in identifying microcalcifications (formed by monosodium urate crystals) in the first metatarsophalangeal joints attacked by gout compared to gray-scale ultrasound images.

**Materials and Methods:**

Thirty-six patients who fulfilled the study inclusion criteria underwent gray-scale ultrasound and MicroPure examinations of the first metatarsophalangeal joints attacked by gout. Static images of the target areas were acquired using gray-scale ultrasound and MicroPure. Two independent and blinded investigators analyzed the images to determine the number of microcalcifications and to score for image quality and artifacts.

**Results:**

The two investigators observed significantly more microcalcifications with MicroPure compared to gray-scale ultrasound (ρ<0.001). The level of agreement between the investigators consistently increased from gray-scale ultrasound to MicroPure imaging (gray-scale interclass correlation coefficient of 0.69 vs. MicroPure interclass correlation coefficient of 0.81). One investigator preferred the MicroPure image quality over gray-scale ultrasound (ρ<0.001), but the other investigator disagreed (ρ<0.001). Both investigators observed fewer artifacts with MicroPure than with gray-scale ultrasound (ρ<0.009).

**Conclusion:**

MicroPure imaging identified significantly more microcalcifications than gray-scale ultrasound.

## Introduction

Gout is one of the most common types of inflammatory arthritis and is caused by the deposition of monosodium urate crystals within joints after chronic hyperuricemia. It affects 1–2% of adults in developed countries and may be increasing in prevalence [1]. The first metatarsophalangeal joint (1^st^ MTP joint) is the most attacked joint; approximately 50–70% of first gout attacks occur here [2]. The gold standard for gout diagnosis depends on identifying the monosodium urate crystals in the joint fluid and confirming the diagnosis with polarizing microscopy [3].

Recently, high-resolution ultrasound (US) has been considered to be a promising tool that could be used in diagnosing and managing gout [4]. High-resolution US has its advantages, such as high spatial resolution, real-time scanning, multi-plane observation, and non-ionizing radiation as well as being a dynamic and non-invasive technique. Furthermore, it can perform repeated measurements in a short time period. Some studies have focused on the usefulness of ultrasound (US) in diagnosing gout and mostly reflected the ultrasonographic characteristics of gouty arthritis. Certain imaging features can facilitate a gouty arthritis diagnosis: the hyperechoic double-contour (DC signs) appearance of the cartilage, a “snowstorm” appearance in the articular cartilage, and hyperechoic deposition (described as bright stippled foci) within the joint cavity [5]. Our study did primarily focus on the last feature. Kuo-Lung Lai [6] *et al*. concluded the sensitivity and specificity of bright stippled foci in gouty joints were 76.9% and 65.4%, respectively. But in our clinic work we found that the visual effect of the bright stippled foci was not satisfactory on gray-scale US. Therefore, how to increase the satisfaction was taken into consideration.

MicroPure (Toshiba America Medical Systems, Tustin, CA) is a US image processing function that is designed to improve the visualization of microcalcifications that can normally be detected but are difficult to visually identify in B-mode images because of the presence of speckle noise and surrounding tissues [7]. This new technique has already been used in evaluating suspicious microcalcifications in breast tissue, and it is possible that MicroPure can be useful in detecting clustered microcalcifications that are inaccessible by B-mode US [8]. Considering its usefulness in detecting microcalcifications, we are attempting to use this new technique to diagnose gouty arthritis.

The aim of this study was to determine whether MicroPure could identify microcalcifications within the joint cavity better than gray-scale US. If so, then in the future, MicroPure might be a useful diagnostic tool in the early stages of gout.

## Materials and Methods

### Patients

The study was approved by the ethics committee of the Shanghai Jiao Tong University Affiliated Sixth People's Hospital, and written consent was obtained from all participants undergoing both MicroPure and gray-scale US imaging.

A total of 36 adult gout patients were recruited for this study in our department from September 2012 to August 2013. The following inclusion criteria were applied: 1) the patients met the standards of American Rheumatism Association guidelines [9] and 2) the 1^st^ MTP joint had a history of acute gouty arthritis. The following exclusion criteria were applied: 1) gout patients without any history of gouty arthritis, 2) chronic gout with the appearance of subcutaneous tophi, and 3) patients with other forms of arthritis, such as rheumatoid arthritis, osteoarthritis, or psoriatic arthritis.

### Methods

The final MicroPure images were generated by superimposing the original B-mode images displayed in blue hues and the extracted microstructures displayed in white spots to identify the locations of the questionable microcalcifications from the gray-scale US images.

All subjects were required to avoid strenuous activities for two hours prior to the examination. All subjects underwent both gray-scale US and MicroPure imaging during the same examination by the same operator. The Aplio 500 TUS-A500 (Toshiba Medical Systems Corporation, Tochigi, Japan) with an 18-MHz broadband linear array probe was used. A total of 42 MTP joints were scanned in 36 patients. The physical parameters were adjusted to obtain the optimizing images and then were kept constant. Transverse and longitudinal static images were obtained from all patients using 2 imaging categories (i.e., gray-scale US, MicroPure). The number of static images obtained for each 1^st^ MTP joint varied from 5 to 10 according to the size and location of the scanned area.

Two experienced investigators who were blinded to the patients' clinical information analyzed all the images. During the study, the investigators were also blinded to one another's image analyses. The number of microcalcifications (e.g., 0, 1, 2, 3, or more) in each image was calculated, and image quality and artifacts were scored on a visual analogue scale (VAS) from 1 (worst) to 10 (best).

### Statistical analysis

The number of microcalcifications in each joint cavity was compared using a paired *t*-test and intra-class correlation coefficient (ICC), while the qualitative scores (i.e., for image quality and artifacts) were compared using a nonparametric Wilcoxon signed-rank test. A ρ value <0.05 indicated statistical significance with a 95% confidence level. All statistical analyses were performed using SPSS 19.0.

## Results

Thirty-six patients (age range, 33–77 years; average age, 55.9 years), including 9 females and 27 males, were enrolled in this study. The duration of gout and the time since last acute attack cited by the patients ranged from 1 to 15 years (mean: 5.9 years) and 0.5–12 months (mean: 5.2 months), respectively. Most of the serum uric acid in patients were abnormal (male: >416 µmol/L, female: >357 µmol/L). Details of the demographic and clinic data are summarized in Table 1.

**Table 1:** Demographic features of patients included in this study.

Variable		Range	Mean ± SD
Age (years)		33–77	55.9±11.3
Maximal serum uric acid (µmol/L)*	male	312–672	510.7±90.5
	female	398–478	443.2±25.7
Most recently serum uric acid (µmol/L)*	male	312–582	432.7±62.4
	female	372–420	394.9±17.2
Duration of gout (years)		1–15	5.9±2.7
Time since last acute attack (months)		0.5–12	5.2±3.7

*: Normal laboratory range is 149–416 µmol/L for males, and 89–357 µmol/L for females.

**Figure 1:**
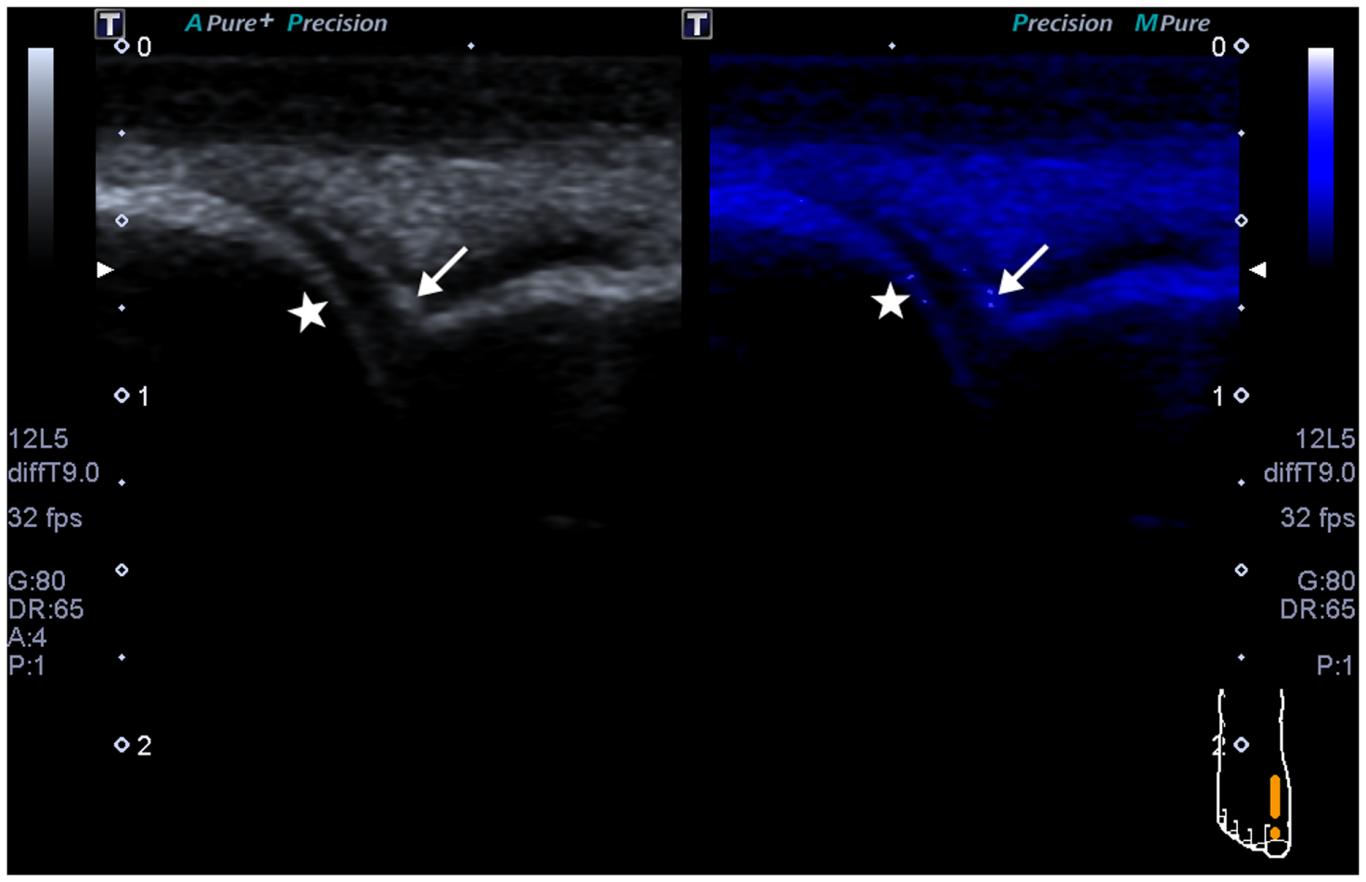
Longitudinal sonograms of the 1st MTP joint. Left: gray-scale US image; Right: MicroPure image. In the articular cavity, there are some microcalcifications (white arrows), which could be observed on both gray-scale US and MicroPure, but some microcalcifications are only observed on MicroPure (white stars).

**Figure 2:**
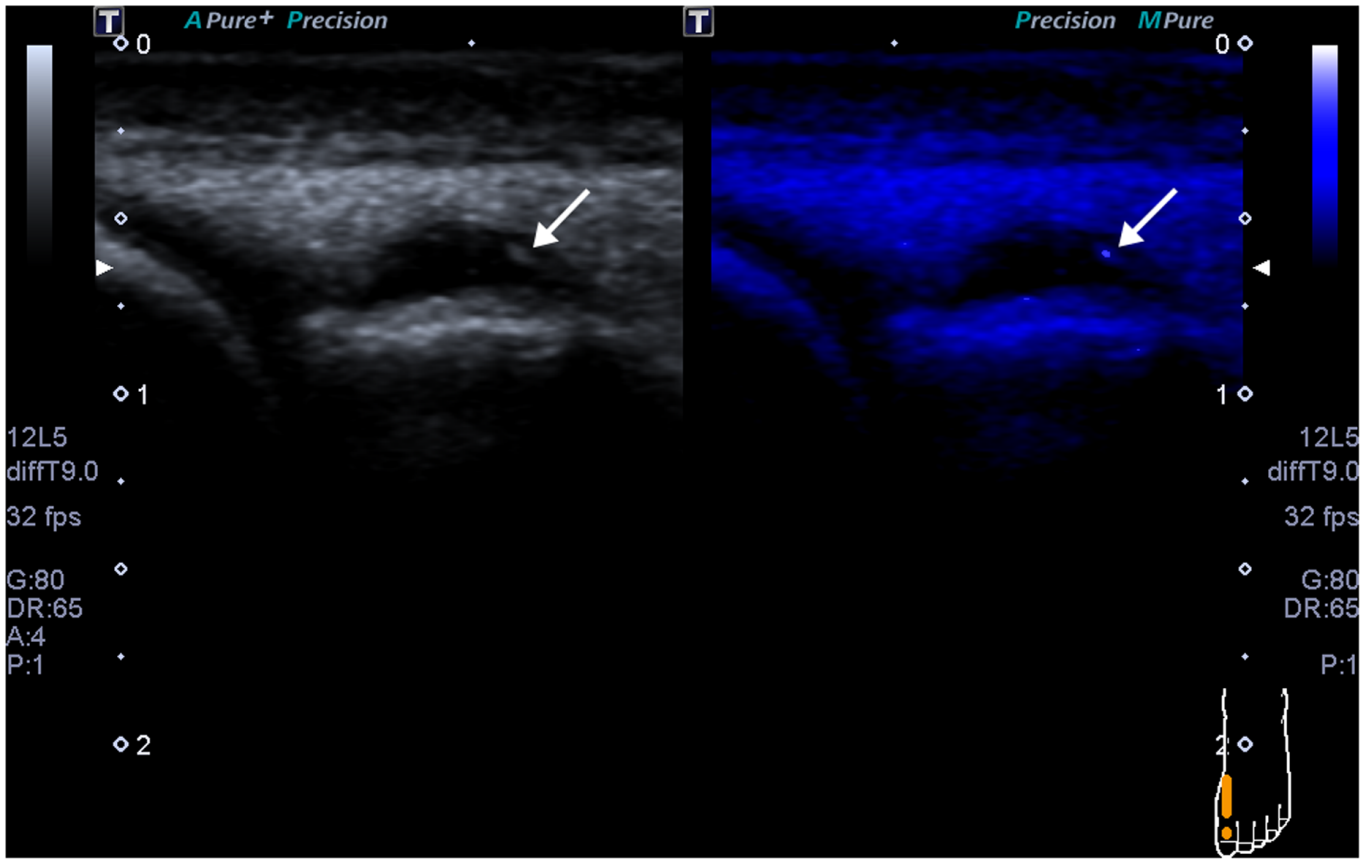
Longitudinal sonograms of the 1st MTP joint. Left: gray-scale US image; Right: MicroPure image. There is a microcalcification in the articular cavity that could not be clearly seen on the gray-scale image (left arrow), but it could be observed on the MicroPure image (right arrow).

Figures 1 and 2 show some examples of static images obtained in gray scale US and MicroPure. Figure 1 shows some microcalcifications (marked with white arrows) in a 1st MTP joint, which could be observed on both gray-scale US and MicroPure, but some microcalcifications are only observed on MicroPure and those microcalcifications are marked with white stars. Figure 2 reflects that there is no bright stippled foci (marked with a white arrow) on gray scale US in the 1st MTP joint. But on MicroPure, the microcalcification (marked with a white arrow) become clear, making a more complete evaluation of the joint.

Both investigators observed more microcalcifications with MicroPure compared to gray-scale US (ρ<0.001). The mean number of calcifications ±SD that the two investigators observed increased from 1.8±0.9 for gray-scale US to 3.9±1.3 for MicroPure. The results for each investigator are shown in Table 2.

**Table 2:** Mean number of microcalcifications observed on the static images.

	Gray-scale US	MicroPure
Investigator 1	1.7±1.0	3.8±1.4
Investigator 2	2.0±0.8	4.0±1.1
Total	1.8±0.9	3.9±1.3

Values are displayed as mean ± SD.

The level of agreement between the investigators consistently increased from the gray-scale US to the MicroPure imaging. The ICC values were 0.69 for gray-scale US and 0.81 for MicroPure.

One of the investigators preferred MicroPure image quality to gray-scale US (ρ<0.001), while the other investigator preferred the image quality of gray-scale US over MicroPure (ρ<0.001). However, regarding artifact evaluations, both investigators observed fewer artifacts with MicroPure than with gray-scale US (ρ<0.009).

## Discussion

The first descriptions of gout can be traced to the beginning of recorded history, but questions still remain regarding the diagnosis of gout [10]. The presence of monosodium urate crystals in joint fluid, confirmed via polarizing microscopy, is considered to be the gold standard for gout diagnosis.

Conventional imaging examinations, including plain radiography, computed tomography (CT), and magnetic resonance imaging (MRI), can supply valuable and plentiful diagnostic advice. However, plain radiography is not useful in the early stages of gout because the monosodium urate crystals are radiolucent, and during this period, there is some non-specific soft tissue swelling and effusion in the inflamed joint, though bone erosions rarely occur. Therefore, plain radiography may show normal findings in the early stages of gout [11]. CT and MRI are more sensitive than plain radiography in detecting early lesions, but they do not show any specific signs [12]. The high costs and inconvenient daily operations of CT and MRI may also limit their use.

Microcalcifications formed by monosodium urate crystals in the articular cavity or on the synovial surface are an important finding for diagnosing gout, and their correct visualization and analysis are important for early detection. In gray-scale US, these microcalcifications manifest as bright dotted foci.

This study focused on the value of MicroPure, a new US image processing technique, for the evaluation of microcalcifications in gouty arthritis. MicroPure extracts the microstructures and emphasizes them using a special filtering method (i.e., the constant false alarm rate). This filter can differentiate between speckle structures and microstructures. The average brightness of the surrounding area is calculated for each pixel, and the difference between the two is the filter output. Thus, a constant false alarm rate filter can detect the locations at which there are characteristic changes compared to the surrounding area [13]. Thus, the extracted points have a high possibility of microcalcifications, and the users can see the microcalcifications without abusing their eyes.

The results of the study are promising, and the mean number of microcalcifications observed on the MicroPure images was increased (3.9±1.3). Conversely, the mean number of microcalcifications observed on gray-scale US was 1.8±0.9.

The investigators had different opinions regarding MicroPure image quality. One investigator preferred MicroPure (ρ<0.001), but the other investigator preferred the image quality of gray-scale US (ρ<0.001). The difference in opinion may be attributed to the investigators' experiences with MicroPure images. Investigators who had viewed MicroPure images prior to the study seemed to adapt to the blue overlay on the MicroPure mode, and this factor may have influenced their readings. The investigator who preferred the quality of the gray-scale US images had never reviewed MicroPure images prior to this study.

The major feature of the artifacts that might have been observed in this study was that bubbles formed as a result of fluid agitation following joint movement [14]. These artifacts may have increased the number of microcalcifications observed on the scanned area. To avoid this problem as much as possible, patients were required to avoid strenuous activities for two hours before the US examination.

In this study, we investigated the value of MicroPure in identifying microcalcifications within the joint cavity compared to gray-scale US. We did not compare MicroPure with other imaging examinations, such as CT and MRI. Future studies will consider these problems. As this study was a pilot study performed on only 36 patients, the sample size was small, and more studies with larger sample sizes are necessary to overcome this limitation and to determine the value of MicroPure imaging in identifying microcalcifications in a clinical setting. Although the two investigators observed fewer artifacts in the MicroPure, the artifacts that were identified can still be confused with microcalcifications. This disadvantage should be ameliorated in the future.

In conclusion, MicroPure identified significantly more microcalcifications than gray-scale US, and the agreement between the investigators was noticeably improved. After this method has been validated in extensive studies, we believe it will be employed specifically in the early diagnosis of gouty arthritis.
